# Significance of targeting *DNMT3A* mutations in AML

**DOI:** 10.1007/s00277-024-05885-8

**Published:** 2024-07-30

**Authors:** Guiqin Huang, Xiaoya Cai, Dengju Li

**Affiliations:** Department of Hematology, Tongji Hospital, Tongji Medical College, Huazhong University of Science and Technology, Wuhan, China

**Keywords:** *DNMT3A* mutations, Acute myeloid leukemia (AML), Targeted therapy, Epigenetics, Oncogenesis

## Abstract

Acute myeloid leukemia (AML) is the most prevalent form of leukemia among adults, characterized by aggressive behavior and significant genetic diversity. Despite decades of reliance on conventional chemotherapy as the mainstay treatment, patients often struggle with achieving remission, experience rapid relapses, and have limited survival prospects. While intensified induction chemotherapy and allogeneic stem cell transplantation have enhanced patient outcomes, these benefits are largely confined to younger AML patients capable of tolerating intensive treatments. DNMT3A, a crucial enzyme responsible for establishing de novo DNA methylation, plays a pivotal role in maintaining the delicate balance between hematopoietic stem cell differentiation and self-renewal, thereby influencing gene expression programs through epigenetic regulation. DNMT3A mutations are the most frequently observed genetic abnormalities in AML, predominantly in older patients, occurring in approximately 20–30% of adult AML cases and over 30% of AML with a normal karyotype. Consequently, the molecular underpinnings and potential therapeutic targets of DNMT3A mutations in AML are currently being thoroughly investigated. This article provides a comprehensive summary and the latest insights into the structure and function of DNMT3A, examines the impact of DNMT3A mutations on the progression and prognosis of AML, and explores potential therapeutic approaches for AML patients harboring DNMT3A mutations.

## Introduction

Acute myeloid leukemia (AML) is a genetically heterogeneous malignancy of immature bone marrow cells due to clonal proliferation and impaired differentiation of hematopoietic stem cells(HSCs), characterized by rapid progression, acquired therapeutic resistance, and vulnerability to relapse [1]. However, progress in AML therapy was relatively slow. The intensive chemotherapy regimen developed in the 1970s consisting of cytarabine and anthracyclines (“7 + 3 regimen”) for AML is still the predominant treatment. Despite updates in the diagnosis and treatment of AML in recent decades, the overall cure rate for patients has not improved significantly [2, 3]. AML patients often confront the dilemma of relapse and refractory remission due probably to the heterogeneity of AML. Therefore, there is an urgent need for personalized treatment regimens tailored to the unique genetic profiles of individual patients, in addition to the conventional chemotherapy approach.

DNA methyltransferase 3 A (DNMT3A) is a de novo methylation enzyme involved in epigenetic regulation and plays a vital role in mammalian development [4]. Recurrent *DNMT3A* mutations were first identified in AML over a decade ago, with mutation frequencies as high as 20–30% [5–7], correlating with increasing age [8]. Studies on the structure, function, and pathogenesis of DNMT3A have shown that the domains of DNMT3A work together to maintain specific DNA methylation profiles that control the gene expression program and affect HSC differentiation and self-renewal [9, 10]. *DNMT3A* mutations facilitate leukemogenesis with other leukemia-related genes [11, 12]. However, there are still no targeted therapies for *DNMT3A* mutant AML, which is related to the unknown mechanism of how DNMT3A mutations affect AML phenotype. Targeting *DNMT3A* mutant AML may require additional prognostic stratification and therapeutic strategies based on molecular mechanisms. In recent years, as research into the pathogenesis of *DNMT3A* mutations in AML has continued, therapeutic strategies targeting *DNMT3A* mutation-dependent DNA methylation and downstream molecules or pathways have emerged, showing great promise for application in the clinical setting.

## DNMT3A and DNMT3A mutations

DNMT3A is a 130 kDa protein encoded by 23 exons on human chromosome 2p23 [13]. The protein expresses in two alternatively spliced isoforms: DNMT3A1 (long) and DNMT3A2 (short) [14]. The essential DNMT3A/3A2-based DNA methylation mechanism is conserved in therian mammals [15].

### Structure and methylation activities of DNMT3A

**Fig. 1:**
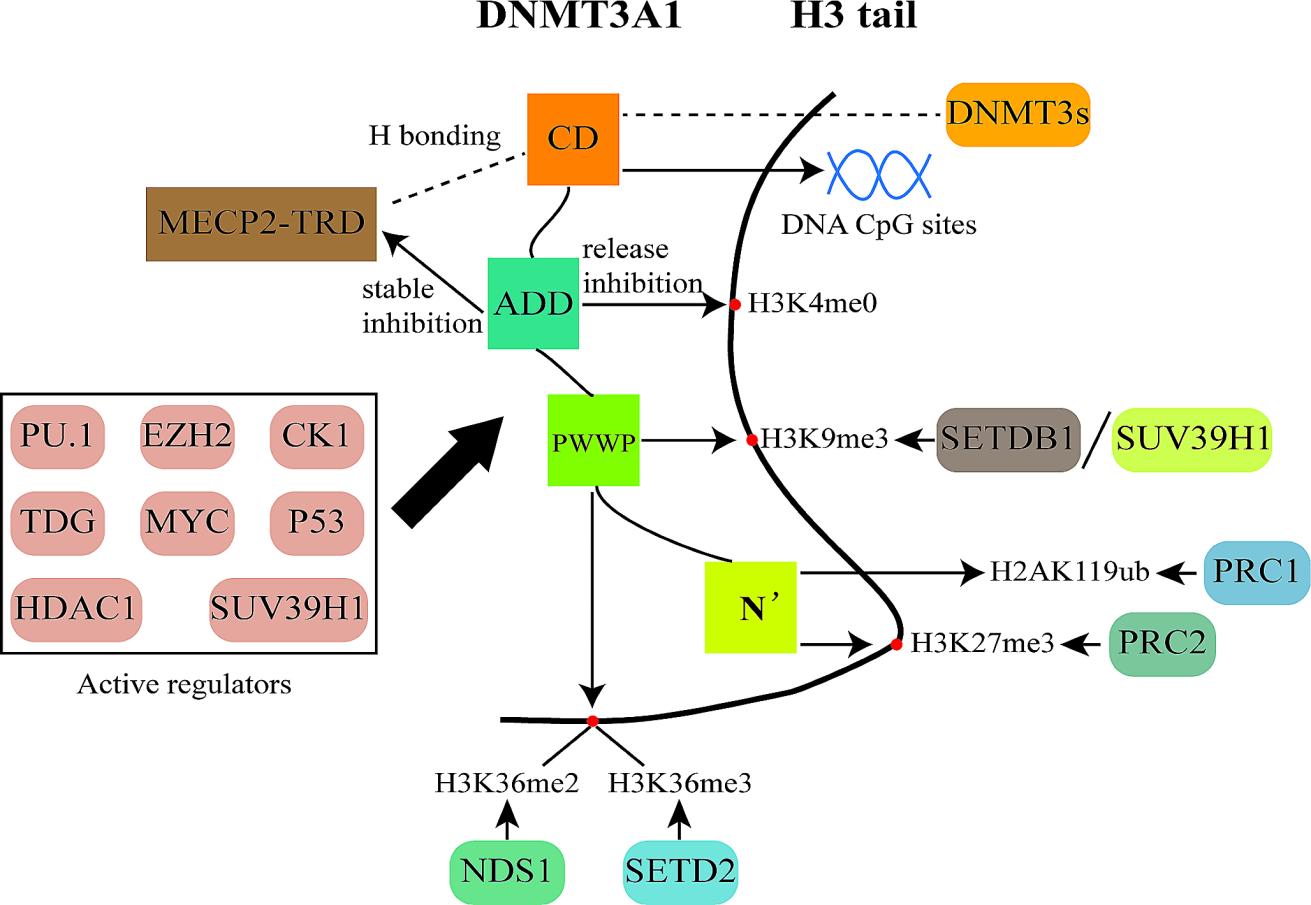
Structure and activity regulation of DNMT3A. The disordered N-terminus interacts with H3Kme3-modified nucleosomes, and H2K119ub-modified nucleosomes, providing two competing mechanisms for DNMT3A recruitment. The PWWP domain of DNMT3A binds to H3K36me2/3 and H3K9me3 marks. Histone H3K36me2 and H3K36me3 form a chromatin platform essential for DNMT3Adependent DNA methylation. DNA CpG methylation and H3K9 methylation are positively correlated with each other in chromatin. The ADD domain recognizes H3K4me0, releasing DNMT3A self-inhibition, and interacts with the MeCP2-TRD to stabilize the self-inhibitory conformation. The CD catalyzes 5’-cytosine methylation within CpG dinucleotides, and binds to itself or other DNMT3 in dimeric, tetrameric, and larger structures. Abbreviations: CD, catalytic domain; ADD, ATRX-DNMT3L-DNMT3A domains; PWWP, Pro-Trp-Trp-Pro domain; N’, The disordered N-terminus

The structure of DNMT3A and its constituent aggregates contribute to related functions(Fig. 1). DNMT3A1 consists of an additional 219 amino acids at the N-terminus compared to DNMT3A2, a regulatory portion composed of the Pro-Trp-Trp-Pro (PWWP) and ATRX-DNMT3L-DNMT3A (ADD) domains, and a conserved catalytic domain at the C-terminus primarily involved in DNA binding and methylation catalysis. The N-terminus of DNMT3A1 is intrinsically disordered and required for DNMT3A1-regulated DNA methylation [16]. The disordered N-terminus and PWWP domains can bind to histone modifications to recruit DNA [17, 18], and the ADD domain regulates enzyme activity by forming contacts with different sites of the catalytic domain of DNMT3A [19, 20]. The ADD domain also interacts with epigenetic factors associated with transcriptional gene silencing, such as some histone-modifying enzymes EZH2, SUV39H1, HDAC1, and the transcription factors p53, PU.1, and MYC [21]. The catalytic domain is a highly conserved catalytic domain at the C-terminus of DNMT3A, which can bind to itself or other DNMT3 in dimeric, tetrameric, and larger structures through two different interfaces in the catalytic domain [22, 23]. DNMT3A structure and regulation of catalysis are already summarized in several excellent reviews [4, 21, 24]. However, how the various domains work together to regulate activity remains challenging. Fundamental questions about how DNMT3A activity is regulated remain unanswered, either through the protein’s intrinsic metastable mechanism or interactions with factors such as nucleotides.

DNMT3A-dependent DNA methylation primarily occurs at CpG dinucleotides [25], with nucleosome positioning as an essential regulatory mechanism. Research shows strong protection of the nucleosomal-bound DNA against methylation, but efficient linker-DNA methylation next to the nucleosome core [26, 27]. DNMT3A has a strong flanking sequence preference for methylating free and linker DNA [28]. In embryonic stem cells, DNMT3A1 prefers to enrich in regions flanked by bivalent promoters marked by H3K4me3 and H3K27me3, often in promoters of developmentally important genes and consistent with the significance of DNMT3A1 in regulating mouse development [16, 29, 30].DNMT3A2 predominantly methylates nuclear body DNA with H3K36me3 [26]. Structural studies have shown that DNMT3A forms a flexible and interdependent interaction network with CpG guanine and flanking residues, ensuring the recognition of efficient methylation of CpG and cytosine in the context of variable flanking sequences [31].DNMT3A constitutes a polymer that also facilitates the recognition of DNA methylation sites. DNA G-quadruplexes may be involved in methylation control [32]. Possible mechanisms of G4-mediated epigenetic regulation may include Dnmt3a sequestration at G4 and or disruption of Dnmt3a oligomerization on the DNA surface [33].DNMT3A/3L heterotetramers have a structural preference for 12 bp-spaced CpG sites and can overcome the preference through their extra DNA interaction patterns during natural DNA methylation [34].

The histone mark recruits DNMT3A and shapes the intergenic DNA methylation landscape. The disordered N-terminus of DNMT3A1 is recruited at the H3K27me3 locus in a PRC2-dependent manner [30, 35] and also interacts with H2AK119ub-modified nucleosomes via a PRC1-mediated mechanism, providing an alternative form of DNMT3A genomic targeting [16, 36]. The histone marker H3K36me2/3 marks the intergenic region and gene body, respectively, forming a chromatin platform that binds to the PWWP region to recruit DNMT3A and is essential for DNMT3A-dependent DNA methylation [37–40]. Two histone marks exert different levels of DNA methylation [40], but how this leads to diverse degrees of DNA methylation is currently unknown, where the differential affinity of DNMT3A to these two marks and the coexistence of other histone marks may play a role. H3K36me3 plays a direct functional role on the stimulation of DNA methylation. H3KC36me3 blocks the hydrogen bond with DNA, resulting in a reduced fit of the H3-tail to the DNA minor groove, which increases the accessibility of linker DNA methylation [26]. In sum, the chromatin localization of DNMT3A1 is likely highly regulated and influenced by the cooperative and competitive binding activities of multiple regulatory domains to diverse epigenetic regulators or transcriptional factors and histone modifications in specific contexts [16]. These studies suggest that DNMT3A may direct CpG methylation through a balance between the recruitment of different reader domains and may underlie the aberrant DNA methylation landscape observed in specific human cancer subtypes and developmental disorders.

The ADD domain recognizes H3K4me0, releasing DNMT3A self-inhibition [19], and interacts with the target recognition domain of the 5mC-binding protein MeCP2 of DNA (MeCP2-TRD) to stabilize the self-inhibitory conformation [20]. So far, further details of the interface and interaction between the regions are unclear. Zhao et al. highlight the importance of structural flexibility for key residues in TRD, generating interest in the rational design of novel DNMT3A inhibitors targeting this region [41]. H3 peptide binding cleft of the ADD domain also mediates the interaction with the MECP2-TRD domain, suggesting that this binding site may have a broader role also in the interactions of DNMT3A with other proteins leading to complex regulation options by competitive and PTM-specific binding [42]. The DNMT3A PWWP domain can be post-translationally modified by phosphorylation of casein kinase CK2, reducing the activity of DNMT3A [43]. The enhancer-binding protein CEBPA blocks DNMT3A from accessing the DNA substrate and inhibits its activities by interacting with DNMT3A N-terminus [44]. In addition, several regulatory proteins also regulate DNMT3A activity, including p53 and thymidine DNA glycosylase (TDG).In the DNMT3A-histone tail-regulatory protein (p53 or TDG) complex, the histone tail primarily isolates DNMT3A from the nucleosome, while p53 or TDG exerts a dominant role in the regulation of DNMT3A activity [45]. However, the relative importance of histone tails and regulatory proteins in the simultaneous coordination of DNMT3A activity remains obscure.

### Overview of *DNMT3A* mutations in AML

*DNMT3A* is mutated as frequently as 20–30% in patients with AML [5–7] and is associated with older age and higher clinical phenotype with higher white blood cell counts [46, 51]. The mutation site of *DNMT3A* in AML differs from other hematologic malignancies, approximately 60% with a specific hotspot point mutation at arginine 882 (R882) at the dimerization interface, most often converted to histidine or cytosine [46, 47]. The R882 mutations exhibit a dominant negative effect [48], significantly inhibiting wild-type DNMT3A (DNMT3A^wt^) by blocking the ability of DNMT3A^wt^ to form an active tetramer [49]. Overexpression of DNMT3A can revert the hypomethylation in Dnmt3a-deficient or R878H mutant cells, while increased levels of DNMT3L, which is underexpressed in AML, can normalize methylation patterns in the Dnmt3aR878H/+ bone marrow [50]. Recent structural studies show that His882 constitutes an inter-subunit contact at the RD interface while Arg882 contacts DNA, contributing two activity centers to the DNMT3A tetramer. The R882H characteristic flanking sequence preferences of DNMT3A are observed in mixed tetrameric complexes containing DNMT3A^wt^ and DNMT3A^R882H^, and they are not diluted by the “averaged” effects of mix or wild-type interfaces. Hence, DNMT3A^R882H^ has a dominant effect on the flanking sequence preferences and other catalytic properties of DNMT3A in samples containing WT and R882H subunits, which may explain its pathogenic influence in the heterozygous state [51]. DNMT3A^wt^ enzyme under conditions that favor non-cooperative kinetic mechanism and DNMT3A^R882H^ variant acquire CpG flanking sequence preference similar to DNMT3B [52]. The introduction of DNMT3B-converting mutations prevents the R882H-/R882C-induced polymerization of DNMT3A and improves substrate accessibility, thus eliminating the dominant-negative impact of the DNMT3A R882 mutations in cells [53]. Higher *DNMT3A* VAFs (> 10%) and the presence of a higher number of coexisting mutations are associated with a higher risk of AML [54]. Approximately 30% of *DNMT3A* mutations exhibit reduced protein stability, which correlates with greater clonal amplification and the development of AML. Regulatory disruption of DNMT3A mediated by the DCAF8 E3 ubiquitin ligase adapter k may explain the mechanism of mutant DNMT3A protein instability [55].

### *DNMT3A* mutations in clonal hematopoiesis

Clonal hematopoiesis (CH) is a condition in which hematopoietic stem cells in normal hematopoietic tissues acquire a potentially cancer-causing gene mutation, and these mutant cells clonally expand in vivo to take up a significant portion of the hematopoietic cells. The incidence of CH increases significantly with age and is called “age-related clonal hematopoiesis” (ARCH). The incidence of CH increases with age and is therefore called “age-related clonal hematopoiesis” (ARCH). Patients with CH have a 5–10 times higher risk of developing hematological malignancies than those without CH, and DNMT3A mutations are the most common genetic event in CH, accounting for 40% of all CH cases, regarded as an early, pro-leukemic event. DNMT3A mutations may lead to enhanced self-renewal of HSCs, which compensates for age-related HSC fatigue but also provides the “first blow” for leukemic transformation. The DNMT3A mutation affects its binding to DNA and chromatin, and its interaction with other proteins, leading to altered DNA methylation patterns and abnormal gene expression [56].

Although clonally expanded HSCs appear to function normally and give rise to a mature, differentiated immune cell lineage that infiltrates virtually all tissues outside the blood compartment, the CH may affect subtle changes in their function, g. inflammatory bowel disease and aplastic anemia) and solid tumors. Here, we limit our discussion to the association of DNMT3A mutation-associated CH with hematological tumors. First, CH can influence tumor pathophysiology through non-tumor cell-autonomous mechanisms. Upon loss of DNMT3A, activated macrophages and mast cells increase the secretion of pro-inflammatory cytokines such as tumor necrosis factor α, IL-6, and CXCL13 [57]. Inflammatory signals associated with the senescent bone marrow microenvironment promote DNMT3-mutant HSC by activating tumor necrosis factor α signaling and interferon γ responses and facilitating their clonal expansion that leads to CH. In addition, extracellular environmental factors, such as bacterial infections, confer a health advantage to Dnmt3-mutant hematopoietic clones [58]. A pivotal study modeled the progression from DNMT3A-driven CH to MPN and eventually AML in mice, suggesting that the transition to expansion of myeloid-restricted progenitors of mutant clones may be an early biomarker [59]. Further studies exploring the link between DNMT3A mutations, CH, inflammation, and immune responses may yield many new and exciting insights with biological and translational implications.

## Effects of *DNMT3A* mutation on AML

### *DNMT3A* mutations affect normal HSC differentiation

**Fig. 2:**
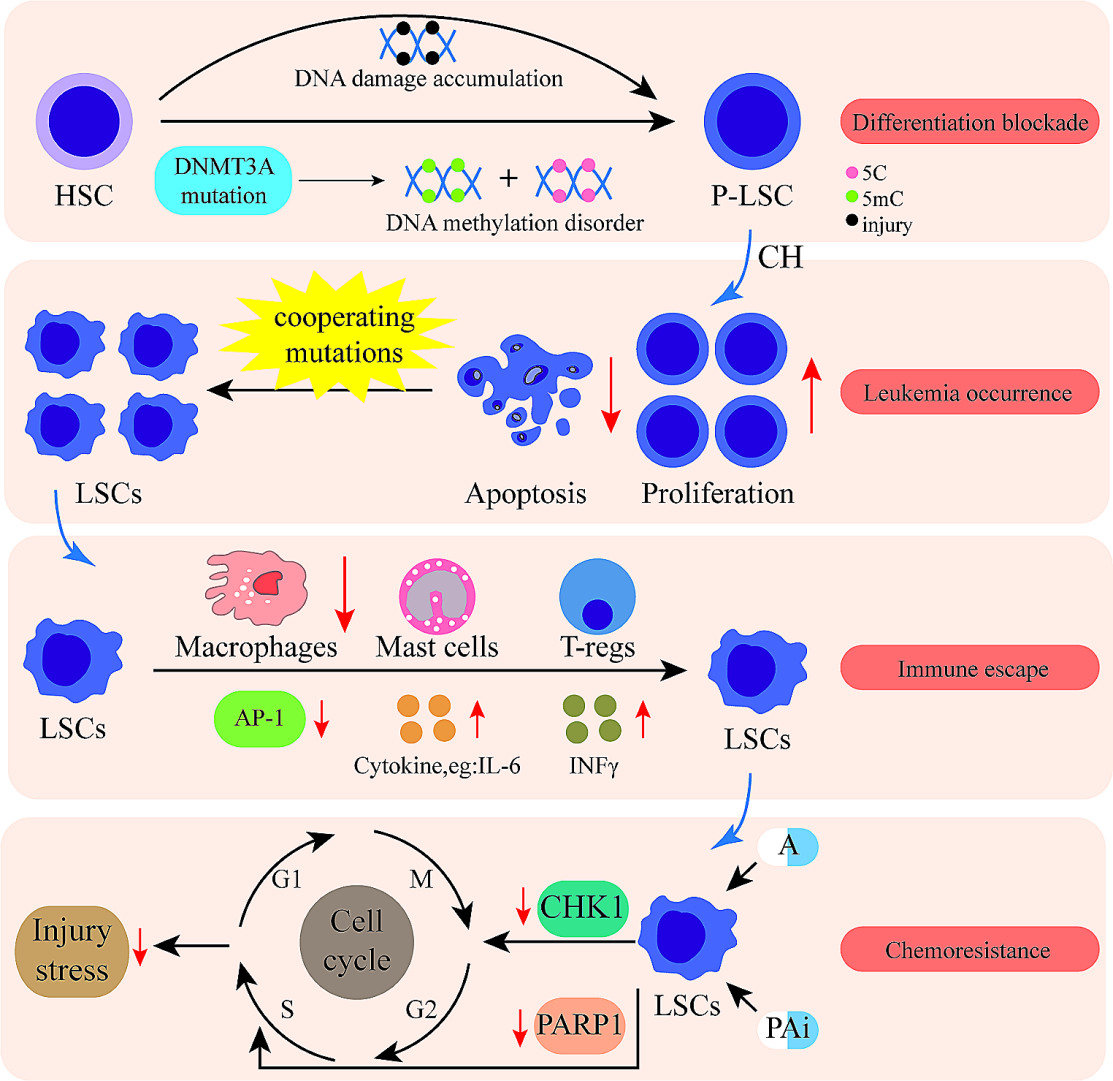
Effects of *DNMT3A* mutation on AML. *DNMT3A* mutant HSCs show abnormal DNA methylation pattern and block normal differentiation; P-LSCs have enhanced self-renewal and diminished apoptosis, and promote AML occurrence under the synergistic effect of co-existing mutations; *DNMT3A* mutant immune cells have abnormal function leading to immune escape of LSCs; *DNMT3A* mutation affects DNA damage repair mechanism causing chemoresistance. Abbreviations: HSC, hematopoietic stem cells; P-LSC, pro-leukemic stem cells; 5 C, non-methylated cytosine; 5mC, 5-methylcytosine; LSCs, leukemic stem cells; A, anthracycline; PAi, PARP inhibitor

*DNMT3A* mutations lead to differentiation blocks in HSCs [60, 61](Fig. 2), while restoring *DNMT3A* expression can partially restore the DNA methylation and gene expression created by the loss of *Dnmt3a* [62]. It may be associated with a downregulation of differentiation-related genes (CEPBA, CEPBE, and PU.1) due to the aberrant interaction of DNMT3A^R882^ with the PRC1 complex at the target site [63]. Recent single-cell multi-omics studies have identified DNMT3A^R882H^ leading to preferential target site hypomethylation of PRC2 components SUZ12 and EZH2, which are hypomethylated motifs enriched in binding motifs of crucial hematopoietic transcription factors [10], exhibiting significant overlap with the previously reported methylation canyon caused by *Dnmt3a* loss [64]. DNA methylation of PRC2 targets can enhance gene silencing [65], so *DNMT3A* mutation-mediated hypomethylation of PRC2 targets may allow mutant progenitor cells to adapt to aberrant reactivation of stem cell maintainers, as seen in PRC2-deficient mouse models [66]. The recent finding that DNMT3A can interact with H2AK119ub-modified nucleosomes in a PRC1-dependent manner as a competitive mechanism mediating DNMT3A targeting towards DNA [16, 36] may explain the preferential hypomethylation of the PRC2 target of mutant DNMT3A and the downregulation of the associated differentiation genes.

Epigenome editing based on Cas9 has shown that the so-called repressive histone modifications H3K9me3 or H3K27me3 are not sufficient to repress gene expression [67], but *Dnmt3a* knockdown (*Dnmt3a*^*−/−*^) comes with an increase in repressive H3K9me3 labeling and an increase in differentiation block [68]. In differentiated tissues or upon SETDB1 removal, a partial subset of the dual domain simultaneously labeled by H3K9me3 and H3K36me3 is disrupted and enriched for activity-enhancing sub-tags [69]. In *DNMT3A* mutant clonal hematopoiesis, different tumor necrosis factor α receptors determine stem cell fitness and lineage output. The genetic loss of the TNFα receptor TNFR1 ablated the selective advantage of mutant HSCs without altering their lineage output, whereas the loss of TNFR2 resulted in the overproduction of mutant myeloid cells without changing HSC fitness. However, it is not clear how this spectral output is regulated [70].

DNMT3A is involved in megakaryocyte-erythroid lineage differentiation and immune cell function. The single-cell multi-omics studies of *Dnmt3a*^*−/−*^ HSCs show a skewed transcriptional initiation toward the red lineage on the granulocyte lineage [9]. In addition, experiments also show that leukemia-initiating cells from *Dnmt3a*^*−/−*^ mice have a megakaryocyte-red lineage progenitor immune phenotype and suppress the corresponding gene expression program [68, 71]. The exact mechanism remains unclear and may be related to preferential hypomethylation of GATA2 targets involved in megakaryocyte-erythroid differentiation [10]. *Dnmt3a*-spermatogonial stem cells also exhibit impaired differentiation during development, associated with the activation of pseudo-enhancers [72]. Specifically, *Dnmt3a* haploinsufficiency impairs the gain of DNA methylation at decommissioned enhancers, while simultaneously and unexpectedly impairing the DNA demethylation of newly activated enhancers in mature human myeloid cells [73]. Thus, enhancer damage may be the cause of the irreversible stem cell genetic program enforced by bone marrow HSCs, resulting in differentiation arrest.

### *DNMT3A* mutations together with co-mutations drive AML development

*DNMT3A* mutations are considered early events in AML [1, 11, 55], usually occur in establishing clonal hematopoiesis, rarely occur alone in AML patients, and are required in conjunction with additional genetic damage to promote AML development [1]. Remarkably, *DNMT3A*^*R882*^ mutations are presumably less frequently observed as disease-initiating mutations but more likely to acquire other mutations and undergo rapid clonal evolution than non-R882 *DNMT3A* mutations based on single-cell sequencing results [11]. *Dnmt3a*^*−/−*^/*DNMT3A*^*mut*^ HSCs have high proliferative activity and live well beyond HSCs/LSCs [74, 75]. Molecular characterization suggests that this in vivo immortalization is associated with a progressive and focal loss of DNA methylation in critical regulatory regions associated with self-renewal genes, producing a highly stereotyped HSC phenotype in which epigenetic features are further supported [76, 77].

*DNMT3A*/*NPM1*, *DNMT3A*/*FLT3*^*ITD*^, *DNMT3A*/*TET2*, *DNMT3A*/*IDH1*/*2* mutations frequently coexist in normal-karyotype AML (NK-AML) patients [12, 54]. DNMT3A and TET2 affect different DNA repair mechanisms [78], remodeling hematopoiesis in opposite ways [79], and TET1 and DNMT3A/B competitive binding synergistically shapes the global DNA methylation pattern in human embryonic stem cells [80]. Early studies demonstrate that *Dnmt3a* haploinsufficiency transforms *Flt3*^*ITD*^ myeloproliferative disease into rapid, spontaneous, complete AML [81]. Subsequently, some researchers bred mouse models expressing *Flt3*^*ITD*^, *Dnmt3a*^*mut*^, and or *Npm1*^*c*^, and evaluated the ability of different combinations of arrangements to induce AML phenotypes. As a result, concurrent expression of *Flt3*^*ITD*^, *Dnmt3a*^*mut*^, and *Npm1*^*c*^ results in a fully penetrant leukemic phenotype, whereas any single or pair of disease alleles either led to longer latency, incompletely penetrant disease or no leukemic phenotype [76]. A novel mechanism of leukemogenesis characterized by the upregulation of hepatic leukemia factor, a specific leukemia transcription factor, is shown to be associated with the simultaneous occurrence of *DNMT3A*^*mut*^, *NPM1*^*mut*^, and *FLT3*^*ITD*^ [82]. Also, by breeding mice with different genotype combinations, *Tet2*^*mut*^*/ Flt3*^*mut*^ and *Dnmt3a*^*mut*^*/ Flt3*^*mut*^ mice do not induce disease at an early stage. In contrast, aggressive transplantation leukemia is observed in *Flt3*^*ITD/wt*^, *Tet2*^*+/−*^, and *Dnmt3a*^*+/−*^ mice within the same time scale, mainly associated with quantitative rather than qualitative differences in gene expression relative to *Tet2*^*mut*^*/ Flt3*^*mut*^ or *Dnmt3a*^*mut*^*/ Flt3*^*mut*^ mice. The heterozygous combination of *FLT3*^*ITD*^, *TET2*, and *DNMT3A* leads to aggressive leukemia [12]. There are distinct mutational cooperativity patterns in AML samples with *DNMT3A* and or *IDH1*/*2* mutations [11]. A misfolded P53 is described in AML blasts that do not harbor mutations in TP53, emphasizing the dynamic equilibrium between wild-type and “pseudo-mutant” conformations of P53. Possible P53 dysfunctions in pre-leukemic hematopoietic stem/progenitor cells carrying *DNMT3A* mutations, may open new avenues for leukemia prevention [83].

### *DNMT3A* mutations disturb immune regulation

In natural immunity, DNMT3A regulates type 1 interferon production by maintaining macrophage histone-lysine deacetylase HDAC9 expression, and *Dnmt3a*^*mut*^ macrophages produce lower levels of type I interferon [84]. *Dnmt3a* haploinsufficiency leads to defective DNA methylation in mature macrophages and aberrant methylation of cell-type specific enhancers [73]. To investigate the mechanism of AML immune escape, one study co-cultures AML cells with macrophages and finds that DNMT3A^mut^ inhibits AML cell pro-inflammatory factor expression suppressing anti-tumor immunity. DNMT3A mutant AML cells regulate macrophage phenotype by suppressing transcription factor AP-1, which attenuates M1 macrophage polarization and resists its killing effect in vitro and in vivo [85].

The DNMT3A constitutes an essential epigenetic mechanism that stabilizes the response pattern of activated T cells. DNMT3-deficient TH 2 cells, TH17 cells, and Treg cells produce aberrant interferon-γ (IFNγ) after restimulation with IL-12 [86]. The critical role of DNMT3A in regulating the homozygous activity of T *Dnmt3a*^*−/−*^ T cells have distinct regions of local hypomethylation, and hypomethylation corresponds to changes in gene expression in several pathways of T cell signaling and differentiation and reveals pathways that control T cell tolerance [87]. The specific impact of *DNMT3A* mutations in allogeneic stem cell transplantation is unclear, although clinical data suggest that donor *DNMT3A* mutations are associated with increased graft-versus-host disease, reduced recurrence, and improved survival [88, 89]. Deleting *Dnmt3a* from CAR-T cells prevents failure and enhances antitumor activity [90]. AML has not benefited from innovative immunotherapies, mainly because of the lack of actionable immune targets. RNA expression of tumor-specific antigens in primary AML samples is associated with mutations in epigenetic modifiers, such as DNMT3A [91]. CD44v6 is aberrantly over-expressed in AML cell lines with *DNMT3A* or *FLT3* mutations, and CD44v6 may be a target for CAR-T in AML patients with *DNMT3A* or *FLT3* mutations [92].

DNMT3A^mut^ affect the expression of downstream-related immune molecules. The miR-196B is hypermethylated and overexpressed in *DNMT3A*^*mut*^ AML and inhibits IKK, IRAK4, NFKB, and MAPK factors in the innate immune signaling pathway [93]. Mast cells lacking *Dnmt3a* exhibit higher cytokine production in response to acute stimulation [57]. AML genotype is associated with a primitive cellular immune phenotype. *DNMT3A* mutations are associated with reduced HLA expression and high levels of other markers (CLIP, PD-L1, TIM-3), and high levels of TIM-3 transcripts in AML primitive cells may be a marker of immune escape [94]. The effects of *DNMT3A* mutations on immune system dysregulation are reviewed in several excellent articles [95, 96].

### *DNMT3A* mutations mediate drug resistance

AML chemoresistance may acquire through genetic mutations [97]. *DNMT3A* mutant cells have increased susceptibility to pharmacologically induced replicative stress, sustained activation of checkpoints within the S phase, impaired recruitment of Poly ADP-ribose polymerase1 (PARP1), and elevated DNA damage. However, *DNMT3A*^*mut*^ cells continue to advance in the cell cycle despite the lag and unresolved DNA damage [98].

Anthracyclines have been used as traditional chemotherapeutic agents in AML for decades and are irreplaceable drugs in AML treatment. In AML clinical trials, cells with DNMT3A^mut^ are less sensitive to anthracyclines [99–101]. Follow-up studies on *Dnmt3a*^*mut*^ hematopoietic models have shown that the relative resistance to anthracyclines is due to abnormal chromatin remodeling and impaired DNA damage perception by blocking the DNA damage response mechanism initiated by checkpoint kinase 1 [76]. *DNMT3A*^*R882H*^ cells have higher NRF2 expression than *DNMT3A*^*wt*^ cells. *DNMT3A*^*R882H*^ cells can respond to erythromycin treatment via the NRF2 / NQO1 pathway [102]. The same group subsequently find that *DNMT3A*^*R882H*^ cells have a higher glucose translocation capacity and a slight inhibition of glycolysis by erythromycin, suggesting a novel mechanism by which the DNMT3A^R882H^ promotes glycolysis through activation of the NRF2/NQO1 pathway [103].

The use of PARP inhibitors as a treatment for AML is since AML driven by repressive transcription factors (AML1-ETO and PML-RARα fusion oncoprotein) is extremely sensitive to PARP inhibition. MLL leukemia, which is even insensitive to PARP inhibition, can be susceptible to PARP inhibitors by reducing the levels of the MLL downstream target HOXA9 protein [104, 105]. Some studies have reported that *DNMT3A* mutant cells favor BRCA1/2-mediated homologous recombination/DNA-PK-mediated non-homologous end-joining due to PARP1 downregulation and reduced PARP1-mediated alternative non-homologous end-joining. Thus, *DNMT3A* mutations are associated with resistance to PARP inhibitors in OTK-transformed hematopoietic cells [78]. However, the coexistence of *DNMT3A* mutations with others or the concatenation of PARP inhibitors with other drugs seems to eliminate the drug resistance caused by *DNMT3A* mutations. The role of PARP inhibitors and their mechanisms have been reported in other literature [106].

### Effect of *DNMT3A* mutations on AML prognosis

The impact on clinical outcomes of AML patients varies by *DNMT3A* mutation types, allele ratio and mutation locations, and the number of sites. Many studies suggest that *DNMT3A* mutations are associated with poor patient prognosis, but the latest 2022 European LeukemiaNet risk stratification does not include *DNMT3A* mutations in the high-risk group. Early clinical studies found no effect of *DNMT3A* mutations on relapse-free survival or overall survival in NK-AML. A negative prognostic impact was found only in the unfavorable NK-AML subgroup of the European LeukemiaNet [46]. The clinical significance of *DNMT3A* mutations appears to be related to age and mutation site. *DNMT3A* mutations in patients younger than 60 years are associated with poorer overall survival, especially among those with intermediate-risk cytogenetics [107]. The *DNMT3A*^*R882*^ mutations are associated with poorer prognosis in older patients, whereas non-R882 *DNMT3A* mutations are associated with poorer prognosis in younger patients [108]. Numerous studies show that R882 mutations show opposite clinical effects to non-R882 mutations, with R882 being detrimental to relapse-free survival and non-R882 being beneficial to overall survival [46, 109]. Interestingly, the frequency of R882 variants in patients with 2 *Dnmt3a* variants is much lower than in the single variants, while patients with 2 *Dnmt3a* variants have significantly shorter event-free survival and overall survival than the single mutant [110].

The combination of *DNMT3A* mutations with different co-mutations has a variable impact on clinical outcomes in AML patients. *DNMT3A* mutations have no significant effect on clinical outcomes in AML patients with *NPM1*^*mut*^ [111], but reduced *NPM1* MRD1 after induction is not predictive of overall survival in patients with *DNMT3A* mutations, and the persistence of *DNMT3A* mutations is associated with a high-risk of relapse [97, 111, 112]. It may be related to the concrete interaction between *DNMT3A* and *NPM1* mutations. *FLT3*^*ITD*^-positive *DNMT3A*^*R882*^ mutation is a poor prognostic factor after allogeneic stem cell transplantation in NK-AML patients [113]. *FLT3* and *NPM1* mutations lead to differential survival in patients with *DNMT3A* mutations and the observation of differentially expressed miRNAs, which may be significant for prognosis [114]. The harmful effects of *FLT3*^*ITD*^ are most clinically relevant in patients with concomitant *NPM1* and *DNMT3A* mutations and are significantly smaller when only *NPM1*^*mut*^ or *DNMT3A*^*mut*^ or neither variant is present [114, 115]. A clinical trial of genomic landscapes of patients with *FLT3*^*mut*^ AML shows that *FLT3*/*DNMT3A*/*NPM1* genotypes do not affect clinical outcomes and suggest that the differential impact of the *FLT3*/*DNMT3A*/*NPM1* genotypes on clinical outcomes may be related to cohort size and characteristics (e.g., age distribution, treatment, genotype, variant type and variant allele frequency) [116]. Patients with *DNMT3A* / *FLT3*^*ITD*^ mutations, in which leukemia switches from an AP-1 regulatory clone at diagnosis to an mTOR signaling-driven clone at relapse [117]. A subgroup of AML patients with *DNMT3A* mutations in both myeloid and lymphoid T-cell compartments, namely lymphoid-myeloid clonal hematopoiesis, has been proposed. Lympho-medullary clonal hematopoiesis can be identified at diagnosis by mutational analysis of T cells and is considered a distinct group of patients with high-risk AML [118]. *DNMT3A* and *TET2* mutations are frequently present in clonal hematopoiesis and are associated with lymph-medullary clonal hematopoiesis LM-CH [118, 119]. The *NPM1*-*DNMT3A*-*NRAS*^*G12/13*^ genotype has an unexpectedly benign prognosis. *DNMT3A* mutations occurring concurrently with IDH2R140 have a significantly lower-than-expected prognosis [1]. Early mutations and amplification of *DNMT3A* and *IDH1*/*2* lead to an increased risk of recurrence [120]. Patients carrying *DNMT3A*^*mut*^, *U2AF1*^*mut*^, and *EZH2*^*mut*^ have worse overall and relapse-free survival rates [121]. Persistent of *DNMT3A*^*mut*^, *TET2*^*mut*^, and *ASXL1*^*mut*^ is frequently present in patients with age-related clonal hematopoiesis and is not associated with an increased relapse rate [122].

## Strategies for targeting *DNMT3A* mutant AML

### Inhibitors of DNMTs

**Fig. 3:**
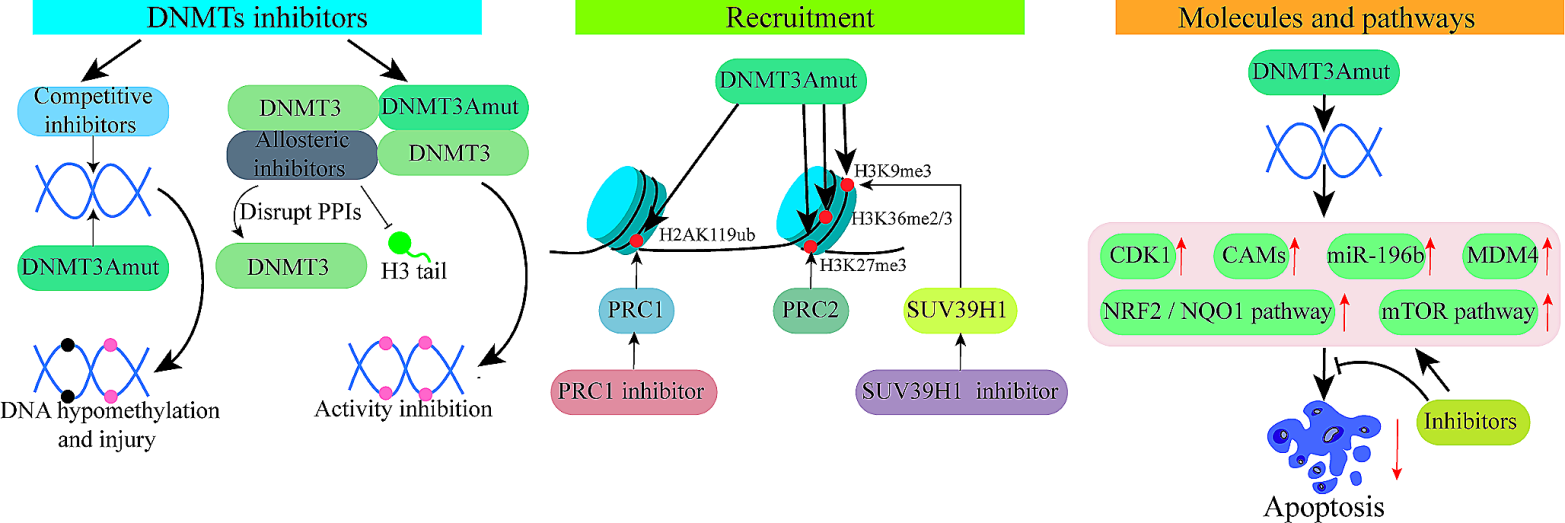
Strategies for targeting DNMT3A mutant AML. Strategies of targeting include: competitive and allosteric inhibitors of DNMTs, prevention of DNMT3A recruitment towards DNA, and block of aberrantly expressed pathways or molecules

Hypomethylating drugs such as azacitidine and decitabine have been used as the basis of low-intensity treatment regimens for AML and MDS(Fig. 3), demonstrating good therapeutic responses in numerous clinical trials alone or combined with other drugs for *DNMT3A*^*mut*^ AML [123–125]. These cytidine analogs bind to DNA and function as covalent competitive inhibitors of DNMTS and DNA damage inducers by forming bulky adducts. However, the targeting mechanism of these hypomethylating drugs for DNMT3 mutant AML is not yet clarified. Moreover, HSCPs expressing *DNMT3A*^*R882H*^ exhibit cell autonomous viral mimicry response as a result of focal DNA hypomethylation at retrotransposon sequences. Administration of azacitidine boost hypomethylation of retrotransposons specifically in *DNMT3A*^*R882H*^ cells and maintain elevated levels of canonical interferon-stimulated genes, thus leading to suppressed protein translation and increased apoptosis [126].

In addition to covalent competitive inhibitors of DNMTS such as azacitidine and decitabine, several non-covalent small molecule DNMT inhibitors are identified and studied [127–129], whose effectiveness against AML remains unreported. One study reports the discovery of a class of DNMT1-selective inhibitors and their improved tolerance in vivo compared to decitabine, yielding a better mouse model of AML [130]. A later study identified two structurally related DNMT3A variant inhibitors of compound1 pyrazolone and compound2 pyridazine by screening a small chemical library. Upon binding to target nucleosomes, these compounds selectively inhibit DNMT3A activity by disrupting the metastable regulation of DNMT3A through H3 tails or PPIs at the tetrameric interface. This inhibition mechanism exists even though DNMT3A compounds with different partner proteins. And these two new compounds may not show the toxicity observed with current therapeutic approaches against DNMT3A. In addition, compound2 pyridazin induces differentiation and apoptosis in distinct myeloid leukemia cell lines, including *DNMT3A*^*R882*^ mutant cells [131].

### Targeting the recruitment of DNMT3A towards DNA

The recruitment of DNMT3A to DNA needs to be mediated by histone modifications, and the production of these histone modifications needs to be catalyzed by a series of enzymes. So targeting these histone-modifying enzymes may regulate the targeting of DNMT3A^mut^ to DNA, thus eliminating the pathogenicity of DNMT3A^mut^. It is important to note that histone modifications as epigenetic modifications, and it is crucial to fully understand the mechanism of action by which histone modifications and DNMT3A regulate each other.

DNMT3A targets DNA with the PRC1/2 complex by two competing mechanisms. DNMT3A^R882H^ leads to preferential hypomethylation of PRC2 targets and disruption of silencing of PRC2 target genes. Conversely, the enhanced interaction of DNMT3A^R882H^ with the PRC1 complex at H2AK119ub-modified target sites leads to the downregulation of differentiation-related genes (CEPBA, CEPBE, and PU.1) [10, 16, 63] .PRC1 regulates AML stem cells and leukemogenesis [132], which makes PRC1 an attractive target for new therapies. The PRC1 inhibitor RB-3 reduces the overall level of H2A ubiquitination and induces differentiation in leukemic cell lines and primary AML samples [133].

The DNMT3A PWWP domain mediates DNA recruitment through recognition of H3K9 methylation [134], and histone methyltransferases that methylate H3K9 are highly conserved and considerably redundant [135]. SETDB1, a hotspot histone methyltransferase that catalyzes H3K9me1/2/3 production, is involved in heterochromatin regulation and gene silencing and has been used as a promising therapeutic site for tumorigenesis [136]. SETDB1 and H3K9 methylation inhibits AML disease progression in vivo by suppressing pre-leukemic genes and relieving the differentiation block to inhibit AML cell growth and self-renewal [137]. Given the vital role of SETDB1 in leukemia, small-molecule inhibitors of the H3K9 methyltransferases may be a therapeutic option. Another histone methyltransferase, SUV39H1, promotes the production of H3K9me3, a tumor-promoting factor in AML, whose loss leads to the activation of the interferon pathway [138]. The SUV39H1 inhibitor, chaetocin, has induced differentiation in AML cells and has synergistic killing effects with other epigenetic drugs [139].

### Targeting aberrantly expressed pathways or molecules

The expression of Exportin1 (XPO1) was found to be elevated in both *DNMT3A*^*R882H*^ AML patients and corresponding cell lines when compared to wild-type counterparts. Selinexor, an inhibitor of XPO1, demonstrated enhanced potency inhibit the proliferation, fostering apoptosis, and impeding the cell cycle progression of *DNMT3A*^*R882H*^ cells when contrasted with wild-type cells. Additionally, selinexor markedly curtailed the growth of subcutaneous tumors in *DNMT3A*^*R882H*^ AML model mice. The study highlighted that selinexor exerts its anti-leukemic effects against *DNMT3A*^*R882H*^ AML by down-regulating the glutathione pathway, the combination of selinexor with Buthionine-(S, R)-sulfoximine (BSO) emerged as a novel therapeutic approach for the treatment of *DNMT3A*^*R882H*^ AML [140]. DNMT3A mutation leads to high expression of nicotinamide adenine dinucleotide (NAD) salvage synthesis pathway key enzyme, nicotinamide-phosphate ribosyltransferase (NAMPT), through DNA hypomethylation, which ultimately leads to abnormal nicotinamide (NAM) metabolism and NAD synthesis, which provides a potential direction for targeting DNMT3A-mutated acute myeloid leukaemia from metabolic level [141]. *DNMT3A*^*R882H*^ upregulates the cyclin-dependent kinase CDK1, which competes with EZH2 for binding to the ADD domain, thereby inhibiting the EZH2 methyltransferase activity and inducing AML [142, 143]. The CDK1-selective inhibitor CGP and the pan-CDK inhibitor flavopiridol cause OCI-AML3 cells to stop in the G2/M phase and induce apoptosis. In addition, the combination of CDK1 inhibitors and conventional chemotherapeutic agents synergistically inhibited proliferation and induced apoptosis in OCI- AML3 cells [144]. The mTOR pathway, another regulator of *HOX* gene, was found to be activated in *DNMT3A* mutant environment, and mTOR inhibitor rapamycin was effective against *DNMT3A* mutant cells in vitro [145]. *DNMT3A* mutant AML cells showed increased adhesion and graphite oxide (GDYO) exhibited specific anti-leukemic effects on *DNMT3A* mutant cells as well as in vivo safety. Mechanistically, GDYO interacts directly with integrin beta 2 and c-type mannose receptor to promote GDYO adhesion and cellular uptake. In addition, GDYO binds to actin and prevents actin polymerization, disrupting the actin cytoskeleton and eventually leading to apoptosis [146]. MDM4, an essential regulator upstream of P53, is overexpressed in NK-AML, particularly in *DNMT3A* mutant samples. The MDM2 / 4 inhibitor ALRN-6924 impairs DNMT3A^wt^/DNMT3A^R882X^ and induces cell cycle arrest through upregulation of p53 target genes, and MDM4 inhibition is a potential target for NK-AML patients with DNMT3A^R882^ [147]. *DNMT3A*^*R882H*^ cells have higher NRF2 expression compared to cells with *DNMT3A*^*wt*^. The NRF2 / NQO1 pathway is activated in mutant cells in response to erythromycin treatment, inhibition of the NRF2 / NQO1 pathway significantly improves the sensitivity of mutant cells to erythromycin, and inhibition of glycolysis re-sensitizes mutant cells to erythromycin [102, 103].

*Dnmt3a*^*−/−*^ HSCs show increased H3K79 methylation relative to *Dnmt3a*^*wt*^ HSCs, with the most significant increase in the DNA methylation canyon, a region highly enriched for genes dysregulated in leukemia. Pharmacological inhibition to the H3K79 methyltransferase DOT1L results in reduced expression of oncogenic canyon-related genes and leads to a dose- and time-dependent inhibition of proliferation, induction of apoptosis, cell cycle arrest and terminal differentiation in *DNMT3A* mutant cell lines in vitro. The DOT1L inhibitor EPZ5676 shows efficacy in both *Dnmt3a*^*mut*^ mice of AML and primary AML patient sample with *DNMT3A*^*mut*^ [148, 149]. Small molecule inhibitors of DOT1L restored *HOXA*/*B* gene repression in vitro and proved effective in *DNMT3A* mutant leukemia [148]. Overexpression of miR-196b in *DNMT3A* mutant AML is essential for maintaining the immature state and survival of leukemic cells by inhibiting Toll-like receptors signaling. Resiquimod, a TLR7/8 agonist, induces dendritic cell-like differentiation in *DNMT3A* mutant AML cells and provides *Dnmt3a*^*mut*^ /*Flt3*^*mut*^ AML mice to provide a survival advantage. In addition, the small molecule bryostatin-1 enhanced resiquimod-mediated AML growth inhibition and differentiation. Thus, overexpression of miR-196b in *DNMT3A* mutant AML creates a novel therapeutic vulnerability by controlling sensitivity to TLR7/8-directed therapies [93]. Donglingin, an enterobactin diterpenoid from the Chinese herb red bean, inhibited *DNMT3A*^*R882*^ mutant leukemic cells at low micromolar concentrations by activating RIPK1-cysteine aspartase-8-cysteine aspartase-3-mediated apoptosis and RIPK1-RIPK3-MLKL-mediated necroptosis. Inhibition of *DNMT3A*^*R882*^ mutant leukemic cells by don glycin can also be observed in vivo [150].

### Targeting *DNMT3A* and cooperating mutations

*DNMT3A* mutations often coexist with other oncogenes in AML, approximately 50% with *NPM1c*, 35–40% with *FLT3*^*ITD*^, followed by about 20% with *IDH1*/*2*, *TET2*, respectively [5–7, 110]. In *Npm1*^*−/−*^/*Dnmt3a*^*−/−*^ mice, leukemia is preceded by an extended period of proliferation and self-renewal of myeloid progenitor cells. This self-renewal can invert by oral administration of a small molecule (VTP-50,469) targeting the MLL50469-Menin chromatin complex [151]. *DNMT3A* and *TET2* mutations remodel hematopoiesis in opposite ways [79], and the competitive binding of TET1 and DNMT3A/B synergistically shapes global DNA methylation patterns in human embryonic stem cells [80]. *TET2* and *DNMT3A* double-mutant cells counteract the resistance of *DNMT3A* mutations and are sensitive to PARP inhibitor treatment [78]. The TLR7/8 agonist assimilate induces dendritic cell-like differentiation with co-stimulatory molecule expression in *DNMT3A* mutant AML cells, providing a survival benefit in *Dnmt3a*^*mut*^ / *Flt3*^*mut*^ AML mice [93]. In *Dnmt3a*^*mut*^ / *Idh2*^*mut*^ HSCs, the epigenome appears dysregulated with the gain of repressive H3K9 trimethylation and loss of H3K9 acetylation and prostaglandin E2 is overproduced. Histone-lysine deacetylase inhibitors rapidly reverse H3K9 methylation/acetylation imbalance and reduce the leukemic burden. HSCs carrying DNMT3A^mut^ and IDH2^mut^ are sensitive to prostaglandin synthesis inhibition. HSCs carrying *Dnmt3a* and *Idh2* mutations are susceptible to prostaglandin synthesis inhibition and differentiation induced by histone-lysine deacetylase [68]. AML with *FLT3*^*ITD*^, *TET2*^*mut*^ and *DNMT3A*^*mut*^ responds to a combination of drugs targeting *Flt3*^*ITD*^, inflammation, and methylation in a mouse model, as well as in a PDX model of AML bearing 3 mutations [68]. The higher risk of AML transformation and worse relapse-free survival in *DNMT3A*^*R882*^ mutant MDS cases is mitigated by coexisting *SF3B1* or *SRSF2* mutations [152].

### Other therapeutic strategies for targeting *DMMT3A* mutant AML

CAR-T therapy shows promising results in the treatment of hematologic malignancies. A current factor currently limiting CAR-T treatment of AML is the selection of specific antigens and complex intrinsic cytogenetic or molecular genetic abnormalities. CD44v6 aberrantly overexpresses in AML cell lines with *DNMT3A*^*mut*^ or *FLT3*^*mut*^. In addition, CD44v6 CAR-T cells show strong anti-leukemic effects on CD44v6 + AML cells, especially AML cells with *DNMT3A*^*mut*^ or *FLT3*^*mut*^ [92].

RNA interference shows therapeutic promise in many cancers. However, no RNAi-based therapies are approved for oncological diseases, probably because serum stability and lack of targeted transport of siRNA limit their systemic antitumor application. One nanocarrier safely transports cargo into AML target cells and is therapeutically active against AML in vitro and in vivo. AML targeting systems consist of internalized anti-CD33-antibody-protamine conjugates that spontaneously form vesicular nanocarriers in an aqueous solution with anionic molecules such as siRNA or ibrutinib-Cy3.5 and cationic free protamine. The siRNA specifically targets the *DNMT3A* mutation and *FLT3*^*ITD*^ mutation in AML, leading to reduced clonal growth of AML cell lines in vitro and inhibiting tumor growth in xenograft cell lines in vivo [153].

There are currently few drugs targeting DNM3A mutant AML and various compounds. Various compounds and therapeutic strategies mainly at the phase of theoretical and experimental exploration are summarized in Table 1.

**Table 1:** Agents and mechanisms of targeted therapy for DNMT3A mutant AML

Agents	Functions	Research stage
Hypomethylating drugs, azacitidine and decitabine	Covalent competitive inhibitors of DNMTS and DNA damage inducers	Clinical therapy [123–125]
GSK3685032	DNMT1-selective inhibitors	Mouse model of AML [130]
Pyrazolone and pyridazine	Structurally related DNMT3A variant inhibitors	Cell lines [131].
PRC1 inhibitor RB-3	Reduce the overall level of H2A ubiquitination	Cell lines and primary AML cells [130]
SUV39H1 inhibitor, chaetocin	Induce CD11b expression and differentiation	cell lines [139]
XPO1 inhibitor, Selinexor	Downregulates the glutathione pathway	mouse model of AML [140]
CGP74514A and flavopiridol	CDK1-selective inhibitor and pan-CDK inhibitor	cell lines [144]
rapamycin	mTOR inhibitor	[145]
Graphite oxide (GDYO)	Bind to actin and prevent actin polymerization, disrupt the actin cytoskeleton	Mouse model of AML [146]
MDM2 / 4 inhibitor ALRN-6924	Induce cell cycle arrest through upregulation of p53 target genes	Primary AML cells [147]
DOT1L inhibitor EPZ5676	Reduce expression of oncogenic canyon-related genes	Mouse model of AML and primary AML cells [148, 149]
Resiquimod	TLR7/8 agonist,	Mouse model of AML [93]
Donglingin, an enterobactin diterpenoid from the Chinese herb red bean	Activate both RIPK1-Caspase-8-Caspase-3-mediated apoptosis and RIPK1-RIPK3-MLKL-mediated necroptosis	Cell lines [150]
VTP-50,469	Target the MLL50469-Menin chromatin complex	Mouse model of AML [151]
CD44v6 CAR-T	specifically lysed CD44v6 + cells while overexpression of CD44v6 in AML cell lines with DNMT3A mutations	Cell lines [92]

## Conclusion

Although molecular mechanisms underlying *DNMT3A* mutant AML are still poorly understood, the impact of *DNMT3A* mutations in AML is evident with the progress of various studies. *DNMT3A* mutations cause aberrant DNA methylation patterns that crosstalk with other layers of epigenetic regulation, altering the normal gene expression profile of HSCs and other somatic cells, and are critical for hematologic malignancy development. However, the relative importance of different DNA methylation sites and how DNMT3A activity targets various motifs are still unclear concerning structural studies of DNMT3A. The multiple targets of DNMT3A emphasize the complexity of its function and the main gaps in our understanding of its regulation. The pathogenic mechanisms of *DNMT3A* mutations in AML require further refinement of the different mutation sites or types. In addition, the synergistic pathogenic effects of *DNMT3A* and coexisting mutations need to explore their molecular mechanisms to provide directions for delineating AML subgroups and refining therapeutic strategies. In recent years, how DNMT3A targets specific sites, how it is recruited to chromatin, and how it interacts with other molecules to regulate methylation activity are slowly revealed with structural studies [16, 34, 45]. The role of *DNMT3A* mutations as pre-leukemic mutations in clonal hematopoiesis have been widely discussed [10, 70, 75]. The relevance of *DNMT3A* mutations to AML phenotypes such as immune escape, drug tolerance, and coexistence of other AML oncogenes has been revealed in numerous clinical trials [12, 94, 102]. As DNMT3A structural studies progress and *DNMT3A* mutation-associated AML clinical phenotypes are studied and understood, various therapeutic strategies have been proposed for *DNMT3A* mutant AML, which may provide patients with more effective options to fight AML.

## Data Availability

No datasets were generated or analysed during the current study.

## Declarations

### Submission declaration and verification

The authors declare that this manuscript has not been published previously, that it is not under consideration for publication elsewhere, that its publication is approved by all authors and tacitly or explicitly by the responsible authorities where the work was carried out, and that, if accepted, it will not be published elsewhere in the same form, in English or in any other language, including electronically without the written consent of the copyright- holder.
